# Does improvement in self-management skills predict improvement in quality of life and depressive symptoms? A prospective study in patients with heart failure up to one year after self-management education

**DOI:** 10.1186/s12872-017-0486-5

**Published:** 2017-02-15

**Authors:** Gunda Musekamp, Michael Schuler, Bettina Seekatz, Jürgen Bengel, Hermann Faller, Karin Meng

**Affiliations:** 1 Department of Medical Psychology and Psychotherapy, Medical Sociology and Rehabilitation Sciences, University of Würzburg, Klinikstr. 3, D-97070 Würzburg, Germany; 2 Institute of Psychology, Department of Rehabilitation Psychology and Psychotherapy, University of Freiburg, Engelbergerstraße 41, D-79085 Freiburg, Germany

**Keywords:** Chronic heart failure, Self-management, Quality of life, Latent change, Cardiac rehabilitation, Patient education

## Abstract

**Background:**

Heart failure (HF) patient education aims to foster patients’ self-management skills. These are assumed to bring about, in turn, improvements in distal outcomes such as quality of life. The purpose of this study was to test the hypothesis that change in self-reported self-management skills observed after participation in self-management education predicts changes in physical and mental quality of life and depressive symptoms up to one year thereafter.

**Methods:**

The sample comprised 342 patients with chronic heart failure, treated in inpatient rehabilitation clinics, who received a heart failure self-management education program. Latent change modelling was used to analyze relationships between both short-term (during inpatient rehabilitation) and intermediate-term (after six months) changes in self-reported self-management skills and both intermediate-term and long-term (after twelve months) changes in physical and mental quality of life and depressive symptoms.

**Results:**

Short-term changes in self-reported self-management skills predicted intermediate-term changes in mental quality of life and long-term changes in physical quality of life. Intermediate-term changes in self-reported self-management skills predicted long-term changes in all outcomes.

**Conclusions:**

These findings support the assumption that improvements in self-management skills may foster improvements in distal outcomes.

## Background

Chronic heart failure (HF) is a common, disabling and fatal medical condition [1]. In view of the aging population it will affect even more people in the future [2]. It requires multidisciplinary management programs including patient education concerning self-care/self-management [3–5]. Self-management includes management of symptoms, treatment, consequences and lifestyle changes implicated by HF and aims to maintain quality of life [6]. For patients with HF, self-monitoring and responding to changes in symptoms are central components of self-management. The situation-specific theory of heart failure self-care [7, 8] states that self-care is a decision-making process, which involves maintenance (treatment adherence and healthy behavior), symptom perception and management (response to symptoms). Self-management interventions proved to be effective with regard to knowledge, self-efficacy, self-management behavior, quality of life, hospitalization, and mortality [9–11]. Thus, self-management is considered the central outcome of such programs and a means to achieve other important outcomes [5, 12]. Self-management can be conceived of as an important proximal outcome of patient education that is a necessary (but not sufficient) prerequisite for the achievement of more distal goals as course of disease, quality of life or social participation [13]. Furthermore, in addition to improving self-management skills during treatment, it is important for patients to sustain and apply the acquired skills after treatment.

Studies have shown that quality of life in HF patients is reduced compared to the general population or patients with other chronic conditions [14–16]. Furthermore, depression is common among patients with HF and worsens the prognosis [17–19]. Therefore, both quality of life and depressive symptoms are considered important distal outcomes in patients with HF.

However, the relations between proximal and distal outcomes of self-management programs have rarely been studied so far. For example, there is no evidence yet that in patients with HF improvement in self-management is actually associated with subsequent improvement in quality of life. Most studies only report separate effects on different proximal and distal outcomes without relating them to each other [10, 11, 20], e. g., without examining whether improvements in knowledge or self-care lead to improvements in quality of life. Two cross-sectional studies showed that self-care behavior (in contrast to self-care confidence) was not associated with higher quality of life in patients with HF [21, 22]. However, these studies did not take into account changes in self-care or quality of life. According to the situation-specific theory of heart failure self-care [7, 8], one study tested whether there were different patterns of change in HF self-care management and whether these were associated with different patterns of change in quality of life over six months [23]. Results show that those who improved in self-care management over time also improved in quality of life. However, the study did not include an intervention and investigated concurrent rather than subsequent changes in outcome variables. In a previous study in patients with different chronic disorders, we showed that short-term changes (before/after inpatient rehabilitation including patient education) in self-reported self-management skills predicted 3-months changes in quality of life and depressive symptoms [24]. The purpose of the present study was to examine whether similar relationships are found in patients with HF and to extend the follow-up period. Particularly, this study investigated whether patients with HF who report an increase in self-reported self-management skills both directly after inpatient rehabilitation including self-management education and after six months show a subsequent increase in quality of life and a decrease in depressive symptoms after both six and twelve months. We hypothesized (1) that the difference of self-reported self-management skills between start and *end of rehabilitation* (duration 3 weeks) predicts the difference of both quality of life and depressive symptoms between start of rehabilitation and follow-up measurements six and twelve months later. We further hypothesized (2) that the difference of self-reported self-management skills between start of rehabilitation and *follow-up after six months* predicts the difference of both quality of life and depressive symptoms between start of rehabilitation and follow-up after twelve months.

## Methods

### Participants and procedure

This is a secondary analysis based on data from a study that evaluated a self-management patient education program for patients with chronic heart failure in inpatient cardiac rehabilitation [25] the results of which were presented elsewhere [26]. A cluster-randomized trial of patients with HF was conducted in four rehabilitation clinics. All participants received a 3-week multidisciplinary inpatient rehabilitation including medical treatment, exercise therapy/physical training, health education, psychological support, relaxation and social counselling as well as a self-management patient education program of different lengths: Patients in the intervention group received a new self-management educational program consisting of five interactive sessions of at least 60 min each provided in small groups. A physician, a nurse, a psychologist and a physiotherapist, respectively, held the interdisciplinary program. Contents were disease and treatment knowledge, self-management behaviors, medication, promotion of physical activity, illness related problems in everyday life, emotional distress and coping strategies. Patients in the control group received a short lecture-based educational program (one lecture of 60 min held by a physician). Contents were disease information and self-management recommendations.

Patients were consecutively included if they had a diagnosis of chronic systolic heart failure (ICD-10: I50) with left ventricular ejection fraction (LVEF) of ≤ 40 and New York Heart Association (NYHA) functional classification II or III. Exclusion criteria were acute events of decompensation, cognitive impairment, insufficient German language ability and severe visual or hearing impairment. Patients completed several standardized patient reported questionnaires at the start (T1) and end (T2) of inpatient rehabilitation as well as after six (T3) and twelve months (T4). Participation in the study was voluntary and all participants provided written informed consent. The study complied with the Declaration of Helsinki and was approved by the ethical review committee of the Faculty of Medicine, University of Würzburg (reference number: 60/11). Detailed information about this trial is presented elsewhere [26]. Of 517 eligible patients 475 comprised the initial study sample. The present analyses are based on those *n* = 342 patients (72%) with data at all four measurement points.

The data of both intervention group (*n* = 178) and control group (*n* = 164) patients were analyzed together, because all received a patient self-management education program. This study does not investigate differences between different education programs but relationships in changes after education programs.

### Measures

We used latent constructs, with selected items from standardized questionnaires serving as indicators.

*Self-management skills* were assessed with the *Skill and technique acquisition* subscale of the Health Education Impact Questionnaire (heiQ^TM^) [27, 28]. It asks for the subjective appraisal of skills and techniques that help manage a chronic disease and related problems (item example: “When I have symptoms, I have skills that help me cope”). It proved to be reliable (Raykov’s Composite Reliability Coefficient = 0.77), uni-dimensional and measurement invariant over time [27, 29]. The items are assessed on a 1-to-4 point Likert scale with higher scores indicating higher skills. All 4 items were used as single indicators for the latent construct *Self-management skills*.

*Quality of life* was assessed using the Kansas City Cardiomyopathy Questionnaire (KCCQ) [30, 31], a disease-specific quality of life measure which proved to be reliable, valid and sensitive to clinical change. The 3 items of the subscale (mental) quality of life (item example: “Over the past 2 weeks, how much has your heart failure limited your enjoyment of life?”) were used as indicators of the latent construct *Mental quality of life*. For the latent construct *Physical quality of life*, 3 (out of 6) items of the physical limitation subscale representing moderate physical activity were chosen as indicators. This subscale measures impairment of everyday activities by HF and thus captures an important aspect of physical quality of life. An item example is: “Please indicate how much you are limited by heart failure (shortness of breath or fatigue) in your ability to do the following activities over the past 2 weeks: Climbing a flight of stairs without stopping”. We did not chose the items covering light physical activity (e.g., dressing or bathing) because we supposed that they might be too “easy” for this sample. All items are scaled from 1 to 5, with higher scores indicating a higher level of quality of life. For the items covering *Physical quality of life*, the response format included a possibility to indicate that the activity was limited or not done for other reasons. Due to this, the sample for the analyses concerning *Physical quality of life* was limited to those *n* = 214 patients who did not indicate this response option in one of these items at any measurement point. The patients who were excluded did not differ from the remaining patients with respect to age, sex, living with a partner, educational level, working status, New York Heart Association (NYHA) class and mean left ventricular ejection fraction (LVEF), respectively (data not shown).

*Depressive symptoms* were assessed with the 2-item-version of the depression module of the Patient Health Questionnaire, PHQ-2 [32]. This reliable (α = 0.83) and valid measure contains two main criteria of depression, diminished interest or pleasure and depressed mood (“Over the past 2 weeks, how often have you been bothered by any of the following problems? Little interest or pleasure in doing things”; “Feeling down, depressed or hopeless”). Items are scaled from 1 to 4, with higher scores indicating higher levels of depressive symptoms. Both items were used as single indicators of the latent construct *Depressive symptoms*.

### Statistical analyses

Statistical analyses were performed similar to Musekamp et al. [24], and detailed description of analytical procedures can be found there. Structural equation modeling techniques were used to investigate the hypotheses.

All models were computed using Mplus version 7.11 [33]. Full information maximum likelihood (FIML) algorithm [34] was used to handle missing data. Proportion of missing data was low (≤7%) for all variables. All models were estimated using a maximum likelihood estimator with robust standard errors [35]. Model fit was assessed based on Chi-square goodness of fit test, Comparative Fit Index (CFI) and Root Mean Square Error of Approximation (RMSEA), with CFI close to or higher than 0.95 and RMSEA close to or lower than 0.06 indicating good model fit [36]. Alpha was set to 0.05 for all analyses.

As preliminary analyses, confirmatory factor analyses (CFA) were applied to test measurement invariance (scalar invariance) over time [37, 38].

Then, to examine the hypotheses, changes in self-reported *Self-management skills* and changes in *Quality of life* and *Depressive symptoms* were modelled applying Latent True Change Modeling [39–42]. This approach allows modelling *inter*individual differences in *intra*individual change in a structural equation framework. Thus, latent (“true”) change can be analysed without measurement error. For judging the size of changes, standardized effect sizes (SES = Mean/SD_1_) were computed for all latent change variables. Standardized estimates for path coefficients are reported for assessment of relations among latent variables. Separate models for the three outcomes (*Physical quality of life, Mental quality of life, Depressive symptoms*) were tested. For identifying the models, the loading of the first item of each factor was fixed to 1, and the first intercept of each factor was fixed to 0. To model indicator-specific effects, correlations between the same indicators (items) at different measurement points were allowed [43]. Two types of models were estimated for all outcomes. The models of the first type use all four measurement points. They predict that changes in self-reported S*elf-management skills* between T1 and T2 predict changes in *Physical quality of life* (model A1), *Mental quality of life* (model B1) and *Depressive symptoms* (model C1) between T1 and T3 and between T1 and T4, respectively (subsequent changes). They further assume the prediction of changes in these outcomes between T1 and T2 (concurrent changes). The models of the second type use the measurement points T1, T3 and T4. These models predict that changes in self-reported S*elf-management skills* between T1 and T3 predict changes in the three outcomes (*Physical quality of life*: model A2, *Mental quality of life*: model B2, *Depressive symptoms*: model C2) between T1 and T4 (subsequent changes), in addition to the prediction of changes between T1 and T3 (concurrent changes).

## Results

### Sample

The sample comprises 342 patients with HF. Table 1 presents the sociodemographic and clinical characteristics. Mean age was 62.0 years (SD = 10.7), 76% were male, and 71% were living with a partner. Educational level was vocational training for most (68%). Most participants were retired (43%) or employed (42%). New York Heart Association (NYHA) class was II for 58%, mean left ventricular ejection fraction (LVEF) was 32.3 (SD = 6.7).

**Table 1:** Sample characteristics (*n* = 342)

	n	%
Male sex	261	76,3
Living with a partner^a^	244	71,3
Educational level^b^		
Vocational training	232	67,8
Technical college	38	11,1
University	19	5,6
Other	40	11,7
Working status^c^		
Employed	143	41,8
Retirement	147	43,0
Unemployed	27	7,9
Other	24	7,0
NYHA II^d^	197	57,6
	M	SD
Age	62,0	10,7
LVEF^c^	32,3	6,7

*NYHA* New York Heart Association class, *LVEF* left ventricular ejection fraction;^a^ missing: *n* = 3.^b^ missing: *n* = 13.^c^ missing: *n* = 1.^d^ missing: *n* = 7

### Preliminary analyses

CFAs testing for configural invariance over time showed good model fit for all variables (CFI > 0.99, RMSEA < 0.05). Metric and scalar invariance models still showed good model fit for all variables (CFI > 0.96, RMSEA < 0.08). There were no misspecifications of parameters as shown by EPC and modification indices. Thus, the assumption of scalar measurement invariance over time was met.

### Latent true change models

#### Model fit

All latent change models yielded good fit with CFI > 0.96 and RMSEA > 0.05 (Table 2). Although the Chi-quare test was significant in some cases, the other fit indices indicated a good approximate fit of the models.

**Table 2:** Goodness of fit summary for latent change models

Outcome	*χ* ^2^	df	*p*	CFI	RMSEA
Model A1: Physical quality of life					
(T1 throughT4)	174.40	151	0.093	0.99	0.03
Model B1: Mental quality of life					
(T1 throughT4)	263.44	151	<0.001	0.97	0.05
Model C1: Depressive symptoms					
(T1 throughT4)	98.24	85	0.155	0.99	0.02
Model A2: Physical quality of life					
(T1, T3, T4)	118.10	110	0.282	1.00	0.02
Model B2: Mental quality of life					
(T1, T3, T4)	150.59	110	0.006	0.99	0.03
Model C2: Depressive symptoms					
(T1, T3, T4)	82.61	67	0.095	0.99	0.03

*CFI* comparative fit index, *RMSEA* root mean square error of approximation

#### Model parameters and effect sizes

Table 3 shows the unstandardized intercepts and factor loadings for the six latent change models. All indicators of one factor have comparable factor loadings, are thus rather homogenous and contribute similarly to the latent constructs.

**Table 3:** Estimated intercepts and unstandardized factor loadings for the latent change models

Model		1: measurement points T1 through T4		2: measurement points T1, T3, T4	
	Item	Intercept	Unstandardized factor loading (SE)	Intercept	Unstandardized factor loading (SE)
A	SM1	0.00*	1.00* (−)	0.00*	1.00* (−)
	SM2	−0.12	1.13 (0.09)	−0.07	1.09 (0.09)
	SM3	−0.19	1.16 (0.10)	0.01	1.07 (0.09)
	SM4	0.82	0.89 (0.09)	1.00	0.81 (0.08)
	pQL1	0.00*	1.00* (−)	0.00*	1.00* (−)
	pQL2	−0.37	1.08 (0.04)	−0.37	1.08 (0.05)
	pQL3	−0.93	1.06 (0.05)	−0.89	1.06 (0.05)
B	SM1	0.00*	1.00* (−)	0.00*	1.00* (−)
	SM2	−0.23	1.16 (0.06)	−0.20	1.14 (0.06)
	SM3	−0.11	1.11 (0.07)	−0.15	1.12 (0.07)
	SM4	0.97	0.83 (0.06)	0.89	0.84 (0.06)
	mQL1	0.00*	1.00* (−)	0.00*	1.00* (−)
	mQL2	−0.41	0.96 (0.04)	−0.43	0.98 (0.04)
	mQL3	0.46	0.89 (0.03)	0.49	0.89 (0.03)
C	SM1	0.00*	1.00* (−)	0.00*	1.00* (−)
	SM2	−0.26	1.17 (0.06)	−0.22	1.14 (0.06)
	SM3	−0.15	1.13 (0.07)	−0.16	1.12 (0.07)
	SM4	0.94	0.84 (0.06)	0.90	0.84 (0.06)
	D1	0.00*	1.00* (−)	0.00*	1.00* (−)
	D2	−0.04	0.83 (0.09)	−0.06	0.85 (0.06)

Fixed parameters are marked with an asterisk. Intercepts and unstandardized factor loadings of same indicators were set equal over measurement points. SM1 to SM4 = Indicators for Self-management skills, pQL1 to pQL3 = Indicators for Physical quality of life, mQL1 to mQL4 = Indicators for Mental quality of life, D1 and D2 = Indicators for Depressive symptoms. SE = standard error

The means, variances and effect sizes (standardized effect size, SES) of latent change variables for all models are shown in table 4. All means of all latent change variables were significantly different from zero, which indicates change in these variables over measurement points. In addition, all variances of all latent change variables were significantly different from zero, indicating interindividual differences in intraindividual change. Effect sizes show that changes in S*elf-management skills* are of medium size, changes in *Physical quality of life* and *Depressive symptoms* are small to medium and changes in *Mental quality of life* are medium to large.

**Table 4:** Unstandardized means and variances and effect sizes of latent change variables

LCV	Model	Mean	Variance	SES
∆SM_2_ − SM_1_	A1	0.26*	0.23^*^	0.48
	B1, C1	0.29*	0.25^*^	0.50
∆SM_3_ − SM_1_	A2	0.27^*^	0.28^*^	0.47
	B2, C2	0.31^*^	0.26^*^	0.53
∆pQL_2_ − pQL_1_	A1	0.23^*^	0.80^*^	0.23
∆pQL_3_ − pQL_1_	A1	0.36^*^	0.97^*^	0.37
	A2	0.35^*^	0.96^*^	0.36
∆pQL_4_ − pQL_1_	A1	0.37^*^	0.91^*^	0.37
	A2	0.37^*^	0.91^*^	0.37
∆mQL_2_ − mQL_1_	B1	0.52^*^	0.31^*^	0.58
∆mQL_3_ − mQL_1_	B1	0.63^*^	0.73^*^	0.71
	B2	0.65^*^	0.73^*^	0.72
∆mQL_4_ − mQL_1_	B1	0.71^*^	0.78^*^	0.80
	B2	0.73^*^	0.78^*^	0.81
∆D_2_ − D_1_	C1	−0.29^*^	0.24^*^	−0.38
∆D_3_ − D_1_	C1	−0.28^*^	0.49^*^	−0.36
	C2	−0.28^*^	0.50^*^	−0.35
∆D_4_ − D_1_	C1	−0.30^*^	0.61^*^	−0.38
	C2	−0.30^*^	0.61^*^	−0.38

*LCV* ∆ = latent change variable, *SM* Self-management skills, *pQL* Physical quality of life, *mQL* Mental quality of life, *D* Depressive symptoms, *SES* Standardized effect size. Subscripted figures represent measurement points

* *p* < .05

#### Path coefficients

**Fig. 1:**
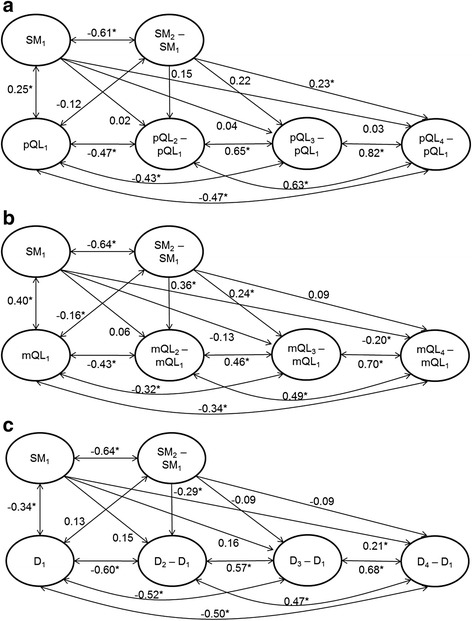
Latent change model (*structural model*) with standardized path coefficients. Predictor Self-management skills (SM), measurement points T1 throughT4. A1: Outcome Physical quality of life (pQL). B1: Outcome Mental quality of life (mQL). C1: Outcome Depressive symptoms (D). Circles represent latent variables, single-headed arrows show the impact of one variable on another, double arrows show correlations allowed between variables. Subscripted figures represent measurement points. *p < 0.05

Figure 1 shows the standardized path coefficients for the structural models A1, B1 and C1 with measurement points T1 through T4. In model A1, interindividual differences in intraindividual change in *Physical quality of life* between T1 and T4 were significantly predicted by change in S*elf-management skills* between T1 and T2 in such a way that increases of *Self-management skills* predicted increases in *Physical quality of life* (β = 0.23). Changes in *Self-management skills* did not predict changes in *Physical quality of life* between T1 and T2 or T1 and T3, respectively. In model B1, increases in *Self-management skills* predicted increases in *Mental quality of life* between T1 and T2 (β = 0.36) and between T1 and T3 (β = 0.24), respectively, but not between T1 and T4. In model C1, increases in *Self-management skills* predicted decreases in *Depressive symptoms* between T1 and T2 (β = −0.29), but not at the later occasions. Additionally, the baseline level of *Self-management skills* predicted changes in both *Mental quality of life* and *Depressive Symptoms* between T1 and T4.

**Fig. 2:**
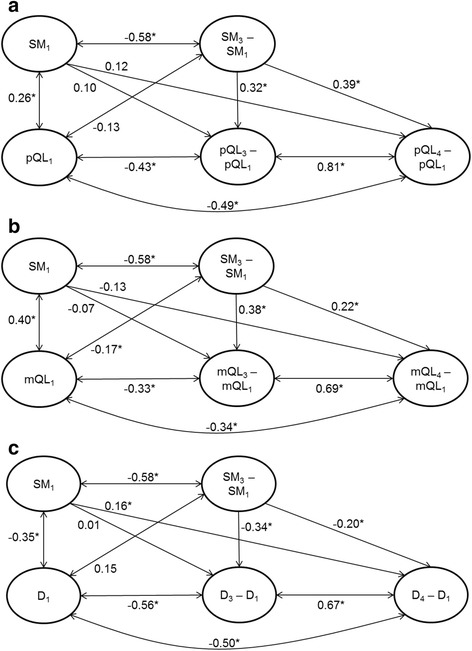
Latent change model (*structural model*) with standardized path coefficients. Predictor Self-management skills (SM), measurement points T1, T3, and T4. A2: Outcome Physical quality of life (pQL). B2: Outcome Mental quality of life (mQL). C2: Outcome Depressive symptoms (D). Circles represent latent variables, single-headed arrows show the impact of one variable on another, double arrows show correlations allowed between variables. Subscripted figures represent measurement points. *p < 0.05

The standardized path coefficients in models A2, B2 and C2 with measurement points T1, T3 and T4 are shown in Fig. 2. In all three models, changes in *Self-management skills* between T1 and T3 predict changes in distal outcomes between T1 and T4. Increases in *Self-management skills* predict increases in *Physical quality of life* (β = 0.39), increases in *Mental quality of life* (β = 0.22), and decreases in *Depressive symptoms* (β = −0.20). Additionally, changes between T1 and T3 in all three outcomes were also predicted by changes in *Self-management skills* (β = 0.32, β = 0.38 and β = −0.34, respectively). Furthermore, the baseline level of *Self-management skills* predicted changes in *Depressive symptoms* between T1 and T4.

## Discussion

To our knowledge, this is the first study that examined the associations between change in self-reported self-management skills and subsequent change in quality of life and depressive symptoms in patients with HF undergoing inpatient rehabilitation including self-management patient education. Results show that changes in self-management skills predict changes in these distal outcomes. Concerning hypothesis 1, results differ between outcomes. Short-term change in self-reported self-management skills (between start and end of rehabilitation) predicted long-term change in physical quality of life (between start of rehabilitation and follow-up twelve months later), but not intermediate-term change (after six months). By contrast, it predicted intermediate-term, but not long-term, change in mental quality of life. Finally, it did neither predict intermediate nor long-term change in depressive symptoms. Thus, hypothesis 1 was only partly confirmed. Regarding hypothesis 2, the picture is consistent: Intermediate-term change in self-reported self-management skills (between start of rehabilitation and follow-up after six months) predicted long-term changes in all three outcomes (between start of rehabilitation and follow-up after twelve months). Thus, hypothesis 2 was confirmed.

The results are in line with Musekamp et al. [24], but not only confirm the prior findings in a HF sample but also expand them with regard to the follow-up period. Although there may be differences in concrete self-management activities between different conditions, increase in self-reported self-management skills is associated with subsequent increase in quality of life and decrease in depressive symptoms regardless of condition. These results are consistent with definitions of self-management and models of patient education in patients with chronic conditions in general suggesting such relationships between self-management skills and quality of life [6, 13]. They are also consistent with the propositions of the situation specific theory of heart failure self-care [8]. Together, they support the importance of self-management in HF patient education [4, 5].

Our results suggest that, in patients with HF, the acquirement of self-management skills during self-management education may have a long-term influence on physical quality of life and an intermediate-term influence on mental quality of life. Thus, improved self-management skills may influence mental quality of life more directly, because they immediately convey a sense of exerting control. Physical quality of life may be influenced in a delayed (but more sustainable) fashion, however, because it takes more time to influence physical conditions. For example, it takes some time until the appropriate reaction to HF symptoms affects the physical condition and the individual takes notice of this improvement. For the long-term changes in mental quality of life and depressive symptoms the baseline level of self-management skills seems a better predictor than the short-term change in self-management skills.

Changes in self-reported self-management skills were not only observed immediately after rehabilitation, but also after six months, with medium effect sizes. Thus, the aim of rehabilitation and self-management patient education to sustain self-management skills in everyday life was reached. Interestingly, change of self-management skills up to six months after rehabilitation seems to influence further change in distal outcomes. Thus, it is not only important to increase self-management skills during self-management education, but also to sustain them afterwards. This emphasizes the importance of fostering self-management skills that can be applied and sustained after treatment. Self-management programs should therefore have a strong focus on everyday life and implement aftercare plans.

However, it remains unclear, whether changes in distal outcomes are initiated by self-management education alone or also by other processes or events coming into effect during the follow-up period. This might for example include events like job changes or retirement.

Further studies should investigate treatment mechanisms of self-management patient education, as they are still unclear [44]. The causal pathway between self-management skills and quality of life needs further examination. It should be examined whether the influence of self-management skills is mediated by actual self-management/self-care behaviors, such as symptom monitoring and responding to symptoms or health behaviors, such as adherence to medication or moderate physical activity [4, 8]. These variables should be included in future models to clarify the effects of self-management skills and other potential predictor variables. Others [45] have investigated the effect of improvement in knowledge after a nurse-led education session on clinical outcomes and found associations with reduced hospital readmissions. On the other hand, knowledge alone may not be sufficient to establish adequate self-care [46]. Therefore, it should be investigated how knowledge and skills work together in establishing adequate self-management behavior and quality of life.

### Limitations

There are some limitations that should be considered. First, while longitudinal associations were examined, causality cannot be proved due to the non-experimental design of our study.

Second, the scale used to assess self-management skills is generic in nature and possibly does not cover all self-management skills important for patients with HF as symptom-monitoring [3]. Thus, our scale might not be specific enough to cover more than general expectations about self-management. Further studies should explore whether specific measures of self-management skills in HF add to prediction of distal outcomes. Third, it was based on self-report and thus susceptible to bias. However, it is difficult to implement objective measures of self-management. Fourth, possibly confounding factors influencing the course of both quality of life and depressive symptoms after self-management education were not taken into account in this study. For example, trait factors like dispositional optimism might influence assessment of both self-management skills and quality of life [47].

## Conclusion

This study adds to previous evidence in that it supports the assumption that self-management skills improved during short- and intermediate-term are associated with intermediate and long-term improvements in both quality of life and depressive symptoms in patients with HF. Thus, relationships between an important proximal outcome of self-management patient education and various important distal outcomes could be demonstrated. The results support the conclusion that patient education interventions for patients with HF should target self-management skills. These should be addressed with a focus on sustainability and suitability in everyday life as some changes became evident only several months after the completion of the educational program. Further studies should investigate additional predictor variables as self-care behavior as well as treatment mechanisms of self-management programs.

## Abbreviations

CFA: Confirmatory factor analysis
CFI: Comparative fit index
D: Depressive symptoms
FIML: Full information maximum likelihood
heiQ: Health education impact questionnaire
HF: Heart failure
KCCQ: Kansas city cardiomyopathy questionnaire
LVEF: Left ventricular ejection fraction
M: Mean
mQL: Mental quality of life
NYHA: New York Heart Association
PHQ-2: Patient health questionnaire depression module 2-item version
pQL: Physical quality of life
RMSEA: Root mean square error of approximation
SD: Standard deviation
SE: Standard error
SES: Standardized effect size
SM: Self-management skills
